# “Include me if you can”—reasons for low enrollment of pediatric patients in a psychopharmacological trial

**DOI:** 10.1186/s13063-021-05119-6

**Published:** 2021-03-01

**Authors:** Larissa Niemeyer, Konstantin Mechler, Jan Buitelaar, Sarah Durston, Bram Gooskens, Bob Oranje, Tobias Banaschewski, Ralf W. Dittmann, Alexander Häge

**Affiliations:** 1 Pediatric Psychopharmacology, Department of Child and Adolescent Psychiatry and Psychotherapy, Central Institute of Mental Health, Medical Faculty Mannheim, University of Heidelberg, J 5, 68159 Mannheim, Germany; 2 Department of Cognitive Neuroscience, Donders Institute for Brain, Cognition and Behavior, Radboud University Nijmegen Medical Center, Nijmegen, The Netherlands; 3 Department of Psychiatry, University Medical Center Utrecht Brain Center, Utrecht, The Netherlands

**Keywords:** Clinical trial, Recruitment, Memantine, Autistic disorder, Obsessive-compulsive disorder

## Abstract

**Background:**

Low recruitment in clinical trials is a common and costly problem which undermines medical research. This study aimed to investigate the challenges faced in recruiting children and adolescents with obsessive-compulsive disorder and autism spectrum disorder for a randomized, double-blind, placebo-controlled clinical trial and to analyze reasons for non-participation. The trial was part of the EU FP7 project TACTICS (Translational Adolescent and Childhood Therapeutic Interventions in Compulsive Syndromes).

**Methods:**

Demographic data on pre-screening patients were collected systematically, including documented reasons for non-participation. Findings were grouped according to content, and descriptive statistical analyses of the data were performed.

**Results:**

In total, *n* = 173 patients were pre-screened for potential participation in the clinical trial. Of these, only five (2.9%) were eventually enrolled. The main reasons for non-inclusion were as follows: failure to meet all inclusion criteria/meeting one or more of the exclusion criteria (*n* = 73; 42.2%), no interest in the trial or trials in general (*n* = 40; 23.1%), and not wanting changes to current therapy/medication (*n* = 14; 8.1%).

**Conclusions:**

The findings from this study add valuable information to the existing knowledge on reasons for low clinical trial recruitment rates in pediatric psychiatric populations. Low enrollment and high exclusion rates raise the question of whether such selective study populations are representative of clinical patient cohorts. Consequently, the generalizability of the results of such trials may be limited. The present findings will be useful in the development of improved recruitment strategies and may guide future research in establishing the measurement of representativeness to ensure enhanced external validity in psychopharmacological clinical trials in pediatric populations.

**Trial registration:**

EudraCT 2014-003080-38. Registered on 14 July 2014.

## Background

The use of psychotropic medication for the treatment of children and adolescents with psychiatric disorders is increasing in Western countries [1–4]. However, most of the prescribed drugs do not have formal regulatory approval for use in this population and are therefore prescribed off-label [5–8]. Several studies have indicated high numbers of off-label prescriptions in numerous European countries. A study by Pagsberg and Thomson described an off-label prescription rate of 30–40% in clinical child and adolescent mental health services in Denmark [9]. The authors reported the lowest rates for drugs approved for treatment of attention deficit hyperactivity disorder (ADHD; 2–3%) and the highest rates for antipsychotics (96%) [9]. A Swiss retrospective study examining off-label prescriptions of psychotropic medications in adolescents in a university psychiatric hospital found that 68% of the medications prescribed in 2014 were prescribed as “off-label” according to age, diagnosis, or dose [10]. This reflected only a minor decrease compared to a rate of 69% off-label prescriptions in 2008 [10]. Clinical trials investigating efficacy and tolerability/safety of psychotropic medications in the population of children and adolescents, which ultimately obtain regulatory approval and market authorization, are therefore urgently needed [11].

According to the American Academy of Child and Adolescent Psychiatry Research Forum, “poor recruitment is the key limiting factor regarding the successful completion of many trials and surveys” [12].

Indeed, low recruitment rates in clinical trials constitute a common and expensive problem, which undermines medical research [13]. Stein et al. conducted a descriptive study concerning barriers to recruitment in clinical trials and found that only 24% (10/41) of the closed studies reviewed by the authors actually met their targeted recruitment goals [13]. Other reports indicated that up to 80% of studies experienced low enrollment and 40–60% did not meet their recruitment goal [14–16]. In a publication describing recruitment issues in a placebo-controlled intervention study on adjunctive vitamin D in the treatment of non-remitted depression, Aucoin et al. reported that out of 148 adult participants who completed screening, only *n* = 24 (16.2%) qualified to participate in the study and only *n* = 9 (6.1%) were successfully enrolled [17].

Research on recruitment issues in trials with children and adolescents is scarce. In an acute care clinical trial, the enrollment rate of pediatric patients was significantly lower than that of adults (40.0% vs. 53.2%) [18]. Hudson et al. conducted a systematic review of studies concerning children with life-threatening illnesses and found that 31% of the included studies recruited less than 50% of potentially eligible participants due to missing contact data, lack of patients’ interest, and negative perceptions concerning potential burdens due to participation [19]. A qualitative study on parents’ agendas in pediatric clinical trial recruitment showed that parents’ trial decisions were mostly based on clinical benefit, child safety, practicalities of participation, and research for the common good [20]. Shilling et al. found “child’s safety” to be the paramount issue for parents [21].

With respect to child and adolescent psychiatry, Bliznak et al. reported that out of *n* = 85 pre-screened children and adolescents with depressive symptoms, the vast majority could not be enrolled in a placebo-controlled clinical registration trial, predominantly due to comprehensive inclusion and exclusion criteria [22]. Emslie et al. concluded in a review of antidepressant clinical trials in children and adolescents that inclusion criteria often resulted in the exclusion of several comorbid disorders and that recruitment in adolescent populations is particularly challenging [23]. In a study investigating the recruitment rate for a randomized controlled trial (RCT) for adolescent bulimia nervosa, a randomization rate of 45% was reported [24]. As pointed out by Kennedy-Martin et al., in order to be clinically useful, the results of RCTs should be generalizable to the real-world patient population that is being investigated, a concept which is referred to as external validity [25]. Studies investigating the proportion of patients with posttraumatic stress disorder (PTSD), aggression, binge-eating disorder (BED), major depressive disorder (MDD), or manic episodes who would qualify for disorder-related RCTs with typical eligibility criteria found alarmingly high exclusion rates: Franco et al. reported that more than 60% of respondents from an overall PTSD sample and more than 70% of respondents seeking treatment for PTSD would have been excluded by one or more exclusion criteria in a typical pharmacological trial [26]. Similarly, the authors of a study on pharmacotherapeutic management of aggression in psychiatric patients described that only 30% of aggressive patients as seen in clinical practice would be eligible to participate in a typical randomized controlled trial [27]. Guerdjikova et al. found that 45.8% of potential subjects with BED were ineligible for study participation because they did not meet entry criteria. Zetin et al. reported an even higher number (91%) of depressive patients presenting for treatment who did not qualify for RCTs when the 11 most common exclusion criteria previously identified in an earlier study were applied [28, 29]. Storosum et al. determined the eligibility of patients with acute manic episodes for a hypothetical but representative RCT, using the most prevalent inclusion and exclusion criteria. They concluded that only 16% of patients’ manic episodes would be eligible for the hypothetical trial.

In a controlled treatment trial of adolescent patients with anorexia nervosa comparing different family treatments and the added effect of fluoxetine vs. placebo, an association was found between participants’/families’ resistance to the study drug (fluoxetine) and poor recruitment. After a recruitment period of 6 months, 47% of patients eligible for the study had refused fluoxetine and only *n* = 20 individuals had been included in the study [30]. Likewise, in a trial involving olanzapine as adjunctive treatment for anorexia nervosa, the authors reported that out of 92 patients approached and asked to participate in the study, only 27 (29%) met full inclusion criteria and only seven (7.6%) were eventually enrolled. The main reasons for study refusal were concerns about medication effects and refusal to contemplate medication as a treatment option [31]. The authors had found similar reasons for poor recruitment (fear of potential adverse events; risk of receiving placebo) in an earlier study in the same target population [32]. In an article discussing obstacles encountered while implementing a randomized clinical trial in a pediatric inpatient psychiatric unit at the Children’s Hospital of Harvard Medical School, the authors stated that parents were often difficult to contact in order to obtain initial consent or data during the study [33].

This is the first study to investigate the recruitment rates of children and adolescents with obsessive-compulsive disorder (OCD) or autism spectrum disorder (ASD) for a randomized, double-blind, placebo-controlled trial and to analyze reasons for non-participation. The examined exploratory ‘Glutamatergic medication in the treatment of Obsessive Compulsive Disorder and Autism Spectrum Disorder’ trial (GOAT) was part of the large, translational project TACTICS (Translational Adolescent and Childhood Therapeutic Interventions in Compulsive Syndromes; http://www.tactics-project.eu/) funded by the European Community’s Seventh Framework Programme (FP7/2007-2013) under grant agreement number 278948, EudraCT Number: 2014-003080-38 [34, 35]. Clinical efficacy (improving symptoms of compulsivity) and tolerability/safety of the glutamatergic agent memantine were investigated in this population at four university-based clinical study sites: (1) Department of Child and Adolescent Psychiatry and Psychotherapy, Central Institute of Mental Health, Mannheim, Germany; (2) Departments of Neuroimaging and Child and Adolescent Psychiatry, Institute of Psychiatry, Psychology and Neuroscience, King’s College London, UK; (3) Department of Child and Adolescent Psychiatry, Brain Center Rudolf, University Medical Center Utrecht, The Netherlands; and (4) Karakter Child and Adolescent Psychiatry, Donders Institute for Brain, Cognition and Behaviour, Radboud University Medical Center, Nijmegen, The Netherlands.

The present study aimed to (i) evaluate the process from pre-screening to inclusion, (ii) systematically analyze reasons for non-participation, (iii) determine factors in trial design or general issues affecting successful recruitment, and (iv) to guide future clinical trials in similar populations by discussing various recruitment strategies and providing evidence and experience-based recommendations.

## Methods

The participating sites pre-screened children and adolescents with obsessive-compulsive disorder (OCD) and/or autism spectrum disorder (ASD) for their eligibility for participation in a multi-national, multi-center, randomized, double-blind, parallel, placebo-controlled trial of glutamatergic medication in the treatment of compulsivity as a cross-disorder trait in OCD and ASD. The overall pre-screening period took place from June 2015 to February 2018, with different time frames for each study center (see Table 1).

**Table 1:** Screening period and patients per study center

Study center	Screening period	Pre-screened patients [n]	%	Enrolled patients [***n***]	%
Mannheim	June 2015–February 2018	88	50.9	2	2.3
Nijmegen	June 2016–February 2018	42	24.3	3	7.0
Utrecht	March 2017–February 2018	43	24.9	0	0
**Total**		173	100	5	2.9

Various recruitment strategies were applied, including the distribution of brochures, informing local office-based child and adolescent psychiatrists, disseminating information on the respective child and adolescent psychiatric department websites as well as oral presentations on the upcoming trial to the medical staff at the respective inpatient and outpatient departments. Contact details of study personnel and information on essential trial content and procedures were provided. Additionally, the local study teams contacted families that had previously given their consent to be informed about available studies.

Potential trial subjects and their parents/legal guardians were asked to participate in the trial by one of the (sub-)investigators and received a letter/informed consent or assent document explaining the trial. They were contacted again after an adequate time interval to consider participation.

Reasons for non-participation for each potential trial subject were documented by three of the four participating sites (Mannheim, Germany; Utrecht and Nijmegen, The Netherlands). Findings were grouped according to content, and descriptive statistical analyses of the data were performed.

## Results

In total, *N* = 173 patients were pre-screened for potential participation in the trial during the 33-month recruitment period. Forty-four (25.4%) were female and *n* = 121 (69.9%) were male; gender was not documented for *n* = 8 (4.6%) pre-screened patients. The mean age was 12.5 years (*n* = 158; range 2–21 years); data on exact age were missing for *n* = 15 subjects (8.7%). *N* = 119 (68.8%) patients with a (suspected) diagnosis of ASD, *n* = 42 (24.3%) with a (suspected) diagnosis of OCD, and *n* = 12 (6.9%) with (suspected) diagnoses of both disorders were pre-screened. Out of the 173 pre-screened patients, only *n* = 5 (2.9%) were eventually enrolled in the trial, leaving *n* = 168 (97.1%) which were either ineligible due to inclusion or exclusion criteria or declined participation. All five enrolled patients had been receiving regular treatment at the respective participating sites prior to trial inclusion.

### Non-eligibility due to inclusion or exclusion criteria

Out of 168 not included patients, *n* = 73 (43.5%) either failed to meet all of the trial inclusion criteria or met one or more of the exclusion criteria (Table 2). Of these, the most commonly reported reasons for non-eligibility were as follows: *n* = 18 (24.7%) patients were either too young or too old to be included (although screening took place in departments of child and adolescent psychiatry, some of the patients were ≥ 18 years old). Per study protocol, age ranges differed for ASD versus OCD patients, ranging from 6 years to 17 years 9 months at initial inclusion for ASD patients and 8 years to 17 years 9 months at initial inclusion for OCD patients. The suspected diagnosis of either ASD or OCD was not confirmed as per study protocol in *n* = 10 (13.7%) pre-screened individuals, and *n* = 9 (12.3%) were already participating in another trial. A diagnosis of either ASD or OCD had to be confirmed on the basis of the Diagnostic and Statistical Manual of Mental Disorders, 5th edition (DSM-5 [36]), according to a structured interview (e.g., DISC [37];) for OCD, or according to the Autism Diagnostic Interview-Revised (ADI-R [38];) for patients with autism.

**Table 2:** Most important inclusion and exclusion criteria of the GOAT trial (for further information see [34])

Most important inclusion criteria	Most important exclusion criteria
- Aged 6 years (ASD patients)/8 years (OCD patients) to 17 years 9 months at initial inclusion - IQ ≥ 70 (based on Wechsler scales, four subtests) - Ability to speak and comprehend the native language of the country in which the assessments take place - DSM-5 diagnosis of OCD (according to a structured interview) and/or ASD (according to ADI-R)	- Intellectual disability (IQ < 70) - Subject has taken another investigational product or taken part in a clinical study with 30 days prior to screening - History of or present clinically relevant somatic acute or chronic disorder that might confound the results of tolerability/safety assessment or would not be in the best interest of the patient

*DSM-5* Diagnostic and Statistical Manual, 5th edition

*ADI-R* Autism Diagnostic Interview-Revised

### Non-participation due to personal reasons

Out of the 168 not included patients and their parents, *n* = 75 (44.6%) declined participation due to personal reasons. Of these, *n* = 40 (53.3%) were not interested in the trial or trials in general, *n* = 14 (18.7%) reported not wanting any changes to their therapy/medication, and *n* = 9 (12.0%) refused to participate because they/their parents disapproved of medication in general. Other personal reasons included perceiving the trial as too burdensome or too complex, “too much going on at home” or being afraid of having blood drawn.

### Non-participation due to general/other reasons

Twenty (11.9%) of the not included patients did not participate due to general reasons. *N* = 6 (30.0%) of these were lost to follow-up. Other reasons were “starting cognitive-behavioral psychotherapy”, medical reasons (e.g., not yet being stable on medication), or patients were currently seeking treatment for another symptom domain/had different treatment requirements.

## Discussion

The present paper reports on systematically collected pre-screening data in a cohort of children and adolescents with (suspected) ASD or OCD who were considered “not eligible” to participate in a multi-national, multi-center, randomized, double-blind, parallel, placebo-controlled trial of glutamatergic medication in the treatment of compulsivity as a cross-disorder trait in OCD and ASD [34]. Our low enrollment rate of only 2.9% is comparable to that of several other psychiatric clinical trials (1.2–7.6% enrollment) [17, 22, 31]. A systematic literature review by McDonald et al. reported an extension of the recruitment period in 53% of 114 trials that recruited participants between 1994 and 2002 in Great Britain, but no consequent improvement of enrollment. In line with these findings, our planned trial enrollment period was extended for one more year with no resulting substantial increase in recruitment [15]. With a focus on ethical considerations, the design of future trials should include a greater consideration of practical feasibility and implementability in order to foster the effective utilization of monetary and human resources.

Although inclusion and exclusion criteria were intended to create an inclusive setting for patient recruitment [34], a considerable subgroup 42% (73/173) of pre-screened patients were not eligible for participation. This was also the case in trials regarding adolescent patients with anorexia nervosa and adolescents with depressive symptoms [22, 31], in which 71% (anorexia nervosa patients) and 75% (depressive symptoms), respectively, failed to meet all of the eligibility criteria defined in the study protocols and were therefore classified as non-eligible.

In any pediatric psychopharmacology clinical trial, the decision to participate involves not only the patient but also the parents/legal guardian. Informed assent/consent is therefore needed from up to three individuals, who may all present specific personal reasons for declining participation.

In line with Guerdjikova et al. [28], who reported a rate of 19.8% regarding lack of interest in trial participation in an adult binge-eating disorder population, we found that 23.1% (40/173) of pre-screened patients and/or their parents were not interested in trial participation or trials in general. Hudson et al. also reported a lack of interest and participants’ perceptions of potential burdens as reasons for non-participation in a systematic review of studies in a pediatric population with life-limiting conditions [19]. It might be argued that patients who benefit from a health care system in a high-income country, with several relatively efficacious treatment options available, might be less willing to participate in clinical trials due to expectations of a small/poor individual benefit/cost ratio. This may differ in countries where trial participation might be the only available and/or affordable treatment option.

In adolescent patients with anorexia nervosa, Lock et al. reported that medication as part of the trial design was significantly associated with poor recruitment, as 47% of eligible individuals refused fluoxetine [30]. Similar findings were reported in the same population in an earlier study, where 70% of eligible patients refused participation due to fears associated with medication effects or refusal to consider medication as a treatment option [31]. In contrast, only 5.2% (9/173) of pre-screened patients and/or their parents in the present trial refused to participate due to the trial medication.

All five enrolled patients had been receiving regular treatment at the respective participating sites prior to trial inclusion. Several recruitment strategies were applied to reach potential participants outside of the participating sites such as distributing brochures, informing local office-based child and adolescent psychiatrists and other institutions in contact with adolescent psychiatric patients as well as disseminating information online. Yet, this did not lead to any additional inclusion. In the authors’ experience this phenomenon is neither singular nor local as “overcoming the walls” of the home trial site to improve recruitment has been a challenge over the last decades at many trial sites in different countries [22].

As laid out in the introduction above, the present trial incorporated mainly four factors which each may account for extra obstacles in recruitment of participants: (i) a pediatric population with (ii) psychiatric disorders and (iii) the investigation of a *psycho*pharmacological agent with (iv) a randomized placebo-controlled trial design. This is reflected by this study’s results which demonstrated reasons for non-participation, expressed by families with children suffering from psychiatric disorders such as no additional capacity (“too much ongoing at home”) and particular health beliefs (“parents disapproved of medication in general”).

Various recruitment strategies are available to overcome these obstacles and have previously been discussed in the literature. Nevertheless, systematic and conclusive evidence in this regard is still missing [12, 13, 39–43].

Table 3 provides an overview of such recruitment strategies commented by the authors in light of their long-term experience in designing and conducting psychopharmacological trials in pediatric populations. A tailored approach to the particular aspects of each individual trial needs to be followed, taking into account the differing conditions and requirements across study sites, given in various regions and countries even in one joint trial, in relation to the respective basic conditions determined, e.g., by health care systems plus regulatory, administrative and ERB agencies.

**Table 3:** Overview of recruitment strategies applied in this trial and rating of their usefulness in pediatric psychopharmacological trials

Recruitment strategy	Pros	Cons	General considerations and recommendations	Rating of usefulness in pediatric psychopharmacological trials^a^
**Contacting local office- or hospital-based psychiatrists/psychotherapists/pediatricians**	- Inexpensive - Targeted	- Can be difficult when many individual offices need to be contacted or visited in person - Trial needs to be carefully explained to colleague/s, therefore complex and time-consuming - If trial potentially interferes with routine care, support by colleague/s is unlikely - Some physicians concerned to ‘lose patient’ to study site after study closure	- Personal contact to colleague/s and collaboration needs to be established and is crucial for effective recruitment - Provide colleague/s with flyers/brochures for distribution to their patients - Aspects of the trial design and integration of participation into regular treatment should be discussed - Easy access and contact to trial team essential (e.g., “hotline”) - Concerns should be taken into serious consideration early on, be discussed openly	**+ + +**
**Contacting self-help/support groups**	- Highly targeted - Useful in diseases with low prevalence - Established network of families interested in progress of research and therapies	- Time consuming	- Personal contact recommended to establish collaboration - Giving presentations, e.g., to groups of parents, about respective disorder, diagnosis and treatment, the current trial and relevant background information should be considered	**+ + +**
**Oral presentations at internal meetings of the department/study site**	- Informative - Targeted	- Time consuming - Short information half-life due to single event character	- Useful to “get the clinical team on board” (at the own institution) and to facilitate collaboration between clinical care and study/research group - Easy access and contact to trial team essential (e.g., “hotline”)	**+ + +**
**Participation in internal regular meetings of clinical team**	- Minimizes risk of missing potential participants - Strengthens established collaboration with clinical team	- Time consuming	- Larger meetings with concise discussion of many patients should be preferred (e.g., admission/discharge planning) - Meetings should be attended regularly (e.g., weekly) and on a long-term basis	**+ + +**
**Keeping a prescreening list**	- Easy - Minimizes risk of losing track of potential participants - Recruitment efforts are documented	- Time-consuming	- Local data protection regulations and ethical guidelines must be followed (e.g., by pseudonymization of personal data)	**+ + +**
**Online and social media ads**	- Easy - Cost-free when untargeted	- Many competing ads - Costly when targeting specific populations	- Post on social media of institution/hospital should be considered - Can easily be repeated on a regular basis	**+ +**
**Information on website of department/hospital/research group**	- Easy - Inexpensive - Targeted	- Needs to be actively accessed (in contrast to ads)	- Information and flyers should be easily visible and accessible for families - Also useful for office-based physicians and other researcher interested in ongoing trials	**+ +**
**Oral presentations at external meetings/conferences**	- Informative - Raises disease awareness	- Time consuming - Untargeted - Short information half-life due to single event character	- Regional and disorder-specific meetings should be preferred - Aspects of the trial design and integration of participation into regular treatment should be discussed - Easy access and contact to trial team essential (e.g., “hotline”)	**+ +**
**Distribution of printed flyers/brochures in community settings/to the general public**	- Easy - Inexpensive - Raises disease awareness	- Untargeted	- Should be considered when recruiting patients with high prevalence disorders or healthy controls - Recommended to identify highly frequented settings for the target population beforehand	**+**
**Newspaper ads/brief articles**	- Mostly easy - Raises disease awareness	- Many competing ads - Untargeted - Can be costly - Potentially, ERB approval needed - At times, content wise mutually exclusive opinions/requests from study management, institutional communication, journal editorial office, ERB	- Should be considered when recruiting patients with high prevalence disorders or healthy controls - Staff should be prepared to face those ‘hurdles’, learn from other sites involved in the trial, be flexible in wording texts.	**+**
**Trial website/blog**	- Informative - Raises disease awareness	- Time and/or cost consuming - Needs to be advertised/made public	- Links to this content should be placed in ads as well as social media and on website of institution/hospital	**+**
**Contacting schools**	- Informative - Raises disease awareness	- Time consuming - Untargeted	- Local regulations and experiences with school authorities vary widely - Can be considered when school problems or disorders with relatively high prevalence (e.g., ADHD) are a focus of the trial or healthy control subjects are part of the trial design	**+**

^a^Rating based on long-term experience of the authors in running clinical pediatric psychopharmacology trials; relative impact on recruitment: + + + high, + + medium, + low

Beyond the specific recruitment strategies presented in Table 3, the authors have found it beneficial to ask patients and their families in treatment for their permission to contact them in the future to inform them about potential participation in an upcoming clinical trial. In order to be effective, this process of keeping a “list of prospective trial participants” should be implemented into routine clinical care while obeying local regulations pertaining to ethical and data protection aspects.

Deciding to participate in psychopharmacological trials, especially those with a placebo-controlled design, and participation itself is complex and potentially burdensome for participants and their families. Therefore, it must be noted that basic principles of professional conversation as applied, for example, in psychotherapy are also fundamental for successful recruitment into clinical trials. This means establishing a trustful relationship when in contact with patients, parents or colleagues, transparently discussing risks and benefits of participation, and communicating in an open and adequate, incl. age-appropriate, manner. Previous research has identified key factors for successful recruitment in clinical trials, i.e., clinicians with a positive attitude to research, cooperation/collaboration of researchers and clinicians, and positive relationships between both the researchers and the recruiting clinicians and the recruiting clinicians and the participant [44]. This context may explain why it has proven to be challenging, in this trial and others, to recruit patients which have not been or are in regular treatment at the trial site since establishing such relationships with patients, their families, and clinicians is much more difficult in these cases.

Furthermore, trial designs in pediatric trials in general should incorporate the needs of both the under-age participants and the parents [45]. If possible, parent representatives should be consulted for key questions in the trial design and recruitment strategies.

These aspects—to be taken into account when designing psychopharmacological trials in pediatric populations—will very likely also apply to clinical trials differing with regard to medical specialty, age group, and intervention/treatment. For example, while not being required in a legal sense, it should also be considered to include relatives of a potential adult trial participant in the informed consent process if agreed to by the potential participant.

Our study represents a post hoc analysis of recruitment problems and reasons for non-inclusion in a pediatric pharmaceutical trial. Apart from recruitment strategies that help to find more patients that could be pre-screened and potentially be enrolled (see Table 3), researchers should also consider whether the rate of ineligibility for their trial could be reduced. Our findings suggest that this could be supported in particular by choosing less restrictive inclusion and exclusion criteria and possibly by reducing the burden for children and their families (taking into account the number of investigations and tests, frequency of visits etc.). Moreover, prescreening lists with various contact data information may also help to not loose patients in the pre-screening process. With regard to reservation against pharmaceutical trials and/or psychotropic medication in general, which had also been identified as a relevant reason for non-inclusion in our study, reducing such reservation of patients and families seems complex and challenging as a broader approach including informative education and psychoeducational programs would be needed.

The observed rate of 97.1% of pre-screened but not enrolled patients in the present study raises the question of participants’ representativeness of real-world patients, and thus of the generalizability of study results from RCTs like the one investigated here. As described in the introduction, several other authors have correspondingly found that only a small proportion of patients with psychiatric conditions would qualify for RCTs targeting their respective disorder [46]. Kennedy-Martin et al. reported in a review of mental health pharmacological studies that RCT samples were highly selected and had a lower risk profile than real-world populations [47]. The authors distinguished between explicit (inclusion/exclusion criteria) and implicit (other issues affecting patient participation) factors influencing external validity. The explicit factors were identified as the “key driver for differences in RCT samples and real-world populations” [47] and consequently as the central reason for the lack of representativeness. Applying this classification to the present trial, 42.2% (73/173) of patients in the study population were not enrolled due to explicit factors. Due to the small number of included participants and missing data on symptom severity or co-morbidities in pre-screened patients, statistical analyses of these factors were not possible. However, several studies in different psychiatric populations have indeed revealed a stronger exclusion rate of patients with higher illness severity, more co-morbidities, and concomitant drug use, suicidal ideation, or substance abuse [28, 47–52].

The influence of implicit factors on generalizability is more difficult to examine. Goedhard et al. reported that adult patients with more severe aggression were less likely to consent to participation, and Kushner et al. found that depressive patients participating in an RCT scored higher on a personality scale assessing preference for novel experiences than did patients treated outside of RCTs [53]. Similarly, we hypothesize that patients who refuse to participate in clinical studies at all, those who disapprove of medication, or those who are medically unstable might have certain personality traits or be more severely ill.

Targeting the issue of low external validity, registry-based randomized controlled trials (rRCTs) have been proposed as a “new clinical trial paradigm” [54]. Compared to conventional RCTs, patient data used for rRCTs are extracted from an existing patient registry, which provides long-term patient data with relatively little effort [55]. It is hoped that rRCTs will enhance generalizability compared to RCTs, by including less-selected patient populations, although there are some critical appraisals emphasizing the limitations of this method [56, 57]. Another relatively new tool to improve the transparency of a priori generalizability of related clinical studies is the “Multivariate Underrepresented Subgroup Identification” (MAGIC) [58]. This algorithm identifies underrepresented subgroups in a set of related studies. He et al. further developed a metric called the Multivariate Generalizability Index for Study Traits (mGIST) in order to quantify the representativeness of real-world patients in clinical trials. The authors concluded that both tools can be used to improve the transparency of design bias in participation selection in clinical research trials [58].

The present findings need to be interpreted in the context of several limitations. As we collected few demographic data (age, gender and disorder) on pre-screened patients, the comparison of enrolled and not-enrolled patients was only possible to a limited degree and we cannot provide comprehensive information regarding the differences between these two groups. However, obtaining data from pre-screening patients is restricted per se, as assent/consent from each patient and parent/legal guardian is a prerequisite for researchers to collect trial-specific information. Furthermore, we only collected pre-screening data in three of the four study centers, resulting in an incomplete dataset. This was due to a delayed recruitment start in the London study center. As the pre-screening data collection took place in different settings and was performed by different researchers, the information obtained might be heterogeneous and partly limited. Moreover, our findings may not be transferable to other disorders, as reasons or motivation for (non-)participation have been found to partially differ depending on symptoms or main diagnoses [27, 47]. The rating of the usefulness of recruitment strategies in pediatric psychopharmacological trials as presented in Table 3 was performed based on the long-term experience of the authors with designing and running clinical pediatric psychopharmacology trials. Despite careful discussion and decision-making based on a relatively large body of experience, this rating nevertheless represents solely the opinion of the authors. With performing a post hoc analysis of recruitment problems, we were able to identify relevant reasons for non-inclusion, but we were not able to test to which extent strategies that appear helpful in this regard will improve recruitment. Further research is therefore needed. Moreover, if recruitment turns out to be problematic in a clinical trial, researchers may consider performing an interim analysis of the recruitment problems during the ongoing recruitment process.

## Conclusions

The present findings add valuable information to the existing knowledge on reasons for low RCT recruitment rates in pediatric psychiatric populations. As in other clinical trials, our enrollment rate was low and the inclusion of patients into the trial was difficult. Study design (inclusion/exclusion criteria) and personal patient and/or parent considerations (lack of interest, refusal to change current medical treatment, disapproval of medication) were the main reasons for non-eligibility/non-participation. The high percentage of pre-screened but not enrolled patients suggests participants’ low representativeness of real-world patients and therefore a potential lack of generalizability of the results of clinical trials of this kind.

The results of this study may inform future trial designs and the effective use of available recruitment strategies while a tailored approach to the particular aspects of each individual trial and to local conditions and requirements needs to be followed. To ensure sufficient external validity in clinical trials, new recruitment strategies and tools to measure representativeness need to be developed, and existing concepts, e.g., registry-based randomized controlled trials and “Multivariate Underrepresented Subgroup Identification” (MAGIC), should be extended and systematically evaluated at the same time. Furthermore, researchers are encouraged to collect and publish detailed findings on recruitment difficulties and reasons for non-participation in clinical trials, in order to develop respective new tools and concepts based on sound data.

## Data Availability

All data generated or analyzed during this study are included in this published article (and its supplementary information files).
